# Jitter-free 40-fs 375-keV electron pulses directly accelerated by an intense laser beam and their application to direct observation of laser pulse propagation in a vacuum

**DOI:** 10.1038/s41598-020-77236-2

**Published:** 2020-11-23

**Authors:** Shunsuke Inoue, Shuji Sakabe, Yoshihide Nakamiya, Masaki Hashida

**Affiliations:** 1 Advanced Research Center for Beam Science, Institute for Chemical Research, Kyoto University, Gokasho, Uji, Kyoto 611-0011 Japan; 2 Department of Physics, Graduate School of Science, Kyoto University, KitashirakawaKyoto, Sakyo 606-8502 Japan

**Keywords:** Ultrafast photonics, Experimental particle physics, Laser-produced plasmas

## Abstract

We report the generation of ultrashort bright electron pulses directly driven by irradiating a solid target with intense femtosecond laser pulses. The duration of electron pulses after compression by a phase rotator composed of permanent magnets was measured as 89 fs via the ponderomotive scattering of electron and laser pulses, which were almost at the compression limit due to the dispersion of the electron optics. The electron pulse compression system consisting of permanent magnets enabled extremely high timing stability between the laser pulse and electron pulse. The long-term RMS arrival time drift was below 14 fs in 4 h, which was limited by the resolution of the current setup. Because there was no time-varying field to generate jitter, the timing jitter was essentially reduced to zero. To demonstrate the capability of the ultrafast electron pulses, we used them to directly visualize laser pulse propagation in a vacuum and perform 2D mapping of the electric fields generated by low-density plasma in real time.

## Introduction

In recent years, ultrafast science based on ultrashort electron pulses with high temporal and spatial resolution has progressed rapidly^1–4^. Electrons with energies of up to several hundred kiloelectron volts, short pulse width, and high brightness have successfully provided information that cannot be obtained using other quantum probes for applications such as ultrafast electron diffraction^5–9^ and electromagnetic field observation^10–13^. Such electron pulses have contributed to numerous scientific advances and can be used to complement ultrashort laser pulses and X-rays^14–17^. For the further development of these applications, and particularly for the observation of faster or irreversible phenomena with high temporal and spatial resolution, it is essential to shorten the electron pulses and further increase the amount of charge. In the development of ultrashort electron pulses, which began with the generation of short pulses of electrons using a DC electron gun^18^, a major challenge is preventing the space charge effect, which increases the pulse width owing to self-generated electric fields^19,20^. By increasing the energy of short pulses of electrons to the megaelectron volt regime, the space charge effect can be suppressed, which has enabled electron pulses of tens of picocoulombs and hundreds of femtoseconds as well as sub-10-fs electron pulses of tens of femtocoulombs^21–23^. A technique has also been developed for minimizing the influence of the space charge effect by placing the specimen or measurement position very close to the DC electron gun^24,25^. Although this method can suppress the increase of electron energy, the space charge effect remains as a limitation. To overcome this problem, the technique of electron pulse compression using an RF cavity as a temporal lens has been introduced with great success, affording sub-picocoulomb electron pulses of several hundred femtoseconds^26,27^. However, when conducting pump–probe experiments with high time resolution using compressed electron pulses, the pulse width is not the only critical parameter, and the timing jitter of the pump pulse and electron probe pulse is equally important. Irrespective of how short a probe pulse can be generated, high time resolution cannot be achieved when observing ultrafast phenomena if the time origin is ambiguous. In recent state-of-the-art devices, for example, phase control using a phase-locking scheme based on passive optical enhancement has enabled the timing jitter to be reduced to approximately 5 fs in 8 min^28,29^. Nonetheless, long-term control has not been achieved. Advanced phase and amplitude control of the RF cavity by active synchronization feedback and passive microwave generation without an optical microwave mixer has successfully enabled stable control with timing jitter of approximately 50 fs in 6 h^30^. However, these methods use high-frequency electric fields for pulse compression, and jitter cannot be completely eliminated. This remains a critical problem when using relatively slow electrons. As an alternative method, pulse compression using a static field instead of RF has been proposed^31–34^. These techniques do not use time-varying electromagnetic fields for electron pulse compression and therefore essentially do not generate timing jitter.

In this work, we report the generation of ultrashort electron pulses with extremely low timing jitter using a static-field compressor. Ultrashort electron pulses with extremely low timing jitter and a short pulse width were achieved by using electrons that were directly accelerated by irradiating a thin aluminium foil with an intense femtosecond laser in conjunction with a pulse compression method involving a phase rotator based on permanent magnets. The measured charge was 16 fC, the FWHM pulse width was 89 fs, and the long-term RMS timing jitter was 14 fs in 4 h. By performing electron pulse compression using only the static field, the timing jitter was essentially zero. Therefore, no complicated or sophisticated control method was required, and we have succeeded in generating electron pulses that are extremely robust against external fluctuations. In addition, by generating the electron pulses from laser plasma, it is not necessary to consider degradation or damage to the photocathode that would limit the number of electrons. The proposed technique has great potential for generating higher-intensity electron pulses using a more powerful laser system^35–37^. In this work, we used a solid foil target to generate electron pulses, rather than using the well-known method of laser-wakefield acceleration (LWFA) as a general laser acceleration scheme^38,39^. LWFA is a very successful method for generating high-energy, quasi-monochromatic, highly directional, ultrashort-pulse electrons. By using LWFA, we believe that it will be possible to efficiently generate electron pulses shorter than the pulse width of laser pulses in the future^40,41^. At present, however, it remains challenging to stabilize the charge, pointing, and energy distribution of electron pulses generated by LWFA over a long period of time, and achieving this requires extremely advanced technology^42–46^, so that we did not use LWFA here. When using a laser-irradiated foil target as an electron source, the electron pulse has a broad energy spectrum that appears to be thermalized and has a wide divergence angle. Therefore, when electrons emitted in a certain direction with a certain energy are extracted as a beam, the laser–electron conversion efficiency is worse in comparison with LWFA. However, if the absorption rate of the laser pulse is stable, that is, if the energy and pointing of the laser pulse are stable and the target foil can be stably supplied, as in this study, it is relatively easy to obtain a stable electron pulse. For this reason, here we used electron pulses generated from foil target, but in the future it will be possible to achieve even shorter pulses and higher brightness with LWFA. To demonstrate the capability of the ultrafast electron pulses, we applied them to the direct visualization of intense laser pulse propagation in a vacuum with a 100-fs time step. Furthermore, the pulses were used to capture the dynamic electric fields generated by low-density plasma originating from the interaction between the intense laser and residual gas in the vacuum chamber.

### System configuration and expected performance

**Figure 1:**
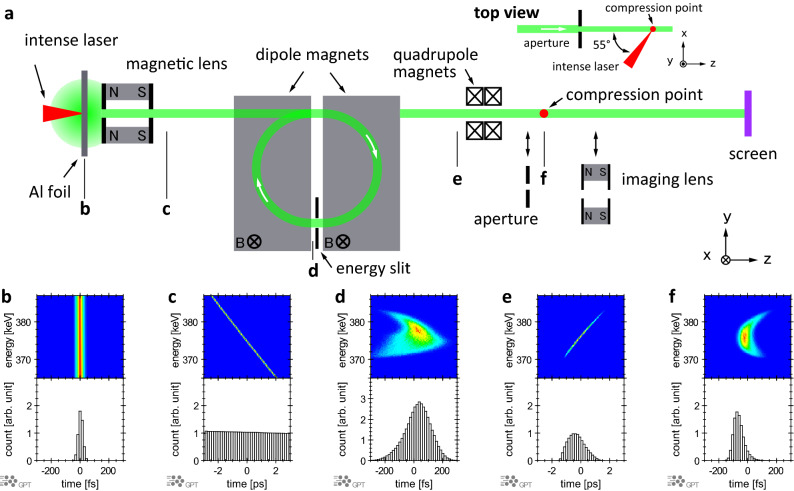
Schematic of experimental setup and simulated temporal characteristics of an electron pulse. (**a**) Schematic of experimental setup for electron pulse acceleration, compression, timing measurements, and pulse width measurements. (**b**–**f**) Simulated longitudinal phase space and histograms for the electrons at each of the positions indicated in (**a**). The initial electron pulse width was assumed to be 40 fs at FWHM and the energy distribution was set to a uniform width of 375 ± 15 keV (**b**). A portion of the electrons passing through the magnetic lens was collimated and automatically chirped with their energy spread (**c**). The collimated electrons were injected into a dipole-type phase rotator to rotate their momentum distribution in longitudinal phase space. The phase space and histogram just after the energy slit of the rotator (**d**), prior to the quadrupole magnets (**e**), and at the compression point (**f**) are also shown.

The experiments were conducted using a Ti:sapphire chirped-pulse amplification (CPA) laser system at Kyoto University^47,48^. Figure 1a shows a schematic of the experimental setup. An intense laser pulse (centre wavelength: 810 nm; pulse width: 40 fs; pulse energy: 400 mJ; repetition rate: 5 Hz; diameter: 50 mm) was split into two pulses. One pulse was used to accelerate the electron pulses, and the other was used to measure the pulse width and timing jitter at the compression point after passing through a delay line. Figure 1b–f show snapshots of the simulated phase-space distribution of electrons in this system calculated using the General Particle Tracer (GPT) code^49^. The electron pulse was accelerated directly by the laser pulse (intensity: 1 × 10^19^ W cm^−2^) striking a piece of aluminium foil with a thickness of 11 μm. Irradiating a solid target with a laser pulse with an intensity greater than 10^18^ W cm^−2^ accelerates electrons with relativistic energy. Electrons are accelerated near the boundary between the solid and the vacuum through various physical mechanisms, namely, J × B heating, vacuum heating, and resonance absorption^50–53^. The laser-accelerated electrons are pushed into the solid thin-film target. A portion of the laser-accelerated electrons pass through the target and are released into the vacuum, while some of the accelerated electrons contribute to overheating of the target and the generation of radiation^54–56^. Here the electrons emitted from the back surface were used as the electron pulse. Because the electron pulse was accelerated during the interaction between the intense laser pulse and the aluminium foil, the width of the electron pulse was considered to be comparable to that of the laser pulse^57^. The aluminium foil target was adjusted using a three-axis motor-driven stage to provide a fresh surface for the laser pulses, which had a repetition rate of up to 5 Hz. The motor-driven stage also corrected the position of the aluminium foil to the focal position of the laser pulse. The RMS position error was reduced to a sufficiently low level (± 3.4 μm) for the Rayleigh length of the laser pulse. The laser irradiation of the aluminium foil resulted in the emission of omnidirectional electron pulses with a broad energy spectrum. These electron pulses were collimated into a quasi-parallel beam by a solenoid-type electron lens composed of a permanent magnet with an aperture of 1-mm diameter. The width of the electron pulse from the laser-irradiated aluminium foil was spread according to the energy dispersion. The collimated electron pulse was injected into a dipole-type phase rotator to rotate its momentum distribution in longitudinal phase space^34^. Because the phase rotator (pulse compressor) does not use a time-varying field, in contrast to an RF cavity^27^ or photoconductive semiconductor switches^58^, it has no influence on the timing drift and very stable pulse compression can be achieved. By adjusting the slit between the compressor magnets, the energy width and centre energy can be selected. In this experiment, an electron pulse with a slit width of 1 mm, a centre energy of 375 keV, and an energy spread of approximately 1% was generated (Fig. 1d). After passing through the pulse compressor, the electron pulse travelled to a compression point, during which the transversal shape was modified using quadrupole magnets driven by an electric current. At the compression point, the electron pulse was compressed to its shortest pulse width, as determined by system aberrations and the pulse width of the electrons upon acceleration (Fig. 1f). A removable aperture of 50-μm diameter was placed 25 mm upstream of the compression point for use during the electron pulse width measurements. A solenoid-type electron lens was positioned downstream of the compression point to image the electrons and measure the timing jitter between the laser and electron pulses. The beam profile of the electron pulse was detected using a fluorescent screen, which was composed of P43 (Gd_2_O_2_S:Tb) coated on titanium foil (foil thickness: 3 μm) to exclude background noise from soft X-rays and low-energy electrons, and an electron-multiplying charge-coupled device (CCD) image sensor^59^. With the exception of the CCD image sensor, the entire system was installed inside a vacuum chamber with a pressure of less than 5 × 10^−2^ Pa.

### Ultrafast electron pulse width measurements

**Figure 2:**
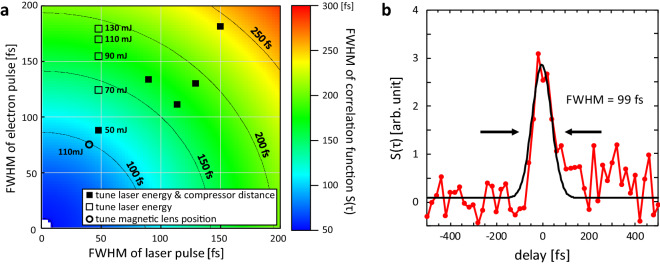
Pulse width measurement based on ponderomotive scattering. (**a**) Electron pulse width inferred from the laser pulse width and the correlation function. The colour scale shows the FWHM of the function S(τ) and maps the result of calculating Eq. (6) using τ_l_ and τ_e_ as variables. (**b**) Cross correlation of the laser pulse and electron pulse at the most compressed condition in (**a**) (open circle). The laser pulse width τ_l_ used for the measurement was 17 fs (40 fs at FWHM), and τ_s_ was 42 fs (99 fs at FWHM).

The pulse width of electrons was measured using the interaction between the laser pulse and electron pulse via the ponderomotive force^20,34,60–63^. Figure 2a shows the pulse width of the electrons inferred from the pulse width of the laser and the correlation function. These results were obtained by adjusting only the laser pulse width, laser pulse energy, and position of the solenoid magnetic lens in the *z*-axis direction. The colour scale in Fig. 2a shows the FWHM of the corresponding function S(τ) (see Methods) and this plot maps the result of calculating Eq. (6) using the laser pulse width τ_l_ and electron pulse width τ_e_ as variables. The laser pulse width and the pulse width of the correlation function S(τ) were obtained experimentally, and τ_e_ was then determined from the colour map.

The solid squares indicate the results obtained when varying the laser pulse width. Because the laser pulse width was adjusted by changing the grating interval of the pulse compressor in the CPA system, both the pulse width for electron acceleration and ponderomotive scattering changed. At constant laser pulse energy, increasing the laser pulse width decreases the intensity of the laser pulse for accelerating the electrons. This results in fewer electrons being generated such that the electron pulse cannot be detected. To avoid this, we adjusted the laser energy along with the pulse width. By adjusting these two parameters, the electron pulse width was compressed to 88 fs (FWHM) for a laser pulse width of 47 fs and laser pulse energy of 50 mJ.

The fluctuation of electron pulse width according to the laser pulse width shown in Fig. 2a (solid squares) originated from changes in both the laser energy and laser pulse width. We assumed that the compression point of the electron pulse varied depending on the acceleration conditions, such as the absorptance of the laser pulse, the amount of pre-plasma generated, and the number of accelerated electrons. In particular, according to the calculations based on the GPT code including the space charge effect, the compression point fluctuated in the *z*-axis direction upon increasing the amount of charge of accelerated electrons. However, quantitative evaluation was difficult because the effects of the laser plasma and foil target cannot be considered in GPT simulations. Nonetheless, from a qualitative perspective, among the reasons for the pulse width variation were the charge amount and the interaction between plasma and electrons. To investigate these effects, the electron pulse width was measured while changing only the laser pulse energy (open squares). The pulse width of the correlation function τ_s_ increased with increasing laser energy owing to the increased number of electrons and variation of the compression point in the *z*-axis direction. To correct for this movement of the compression point, we adjusted the position of the magnetic lens in the *z*-axis direction, which is equivalent to changing the position of the compression point. This resulted in the data point indicated by the open circle in Fig. 2a, which corresponded to a laser energy of 110 mJ.

**Figure 3:**
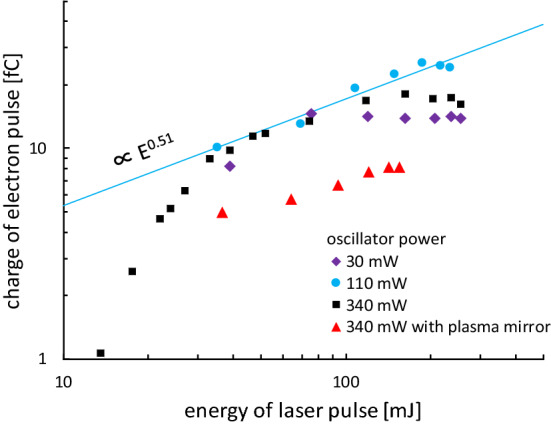
Laser pulse energy and contrast ratio dependence of the charge of the electron pulse. The charge of electron pulses with an energy of 375 keV detected using the fluorescent screen is plotted. To increase the charge, the pulse contrast ratio of the driving laser was adjusted by changing the input energy from the oscillator to the amplifier in the CPA laser system. For the input energy of 340 mW, the contrast ratio was 10^−7^. The input energies of 30 and 110 mW correspond to changing the contrast ratio by 11-fold and 3.1-fold compared with contrast ratio at an input energy of 340 mW. The results obtained using a plasma mirror^48^, which improved the contrast ratio from 10^−7^ to 10^−9^, are also shown (see Methods).

To increase the number of electrons, the contrast ratio between the pedestal pulse and the intense laser was adjusted by changing the power of the oscillator (Fig. 3, see Methods). It is assumed that the contrast ratio at which most electrons are emitted depends on the laser intensity and energy and the type and thickness of the thin foil target, because these parameters affect the properties of the preformed plasma and the absorptance of the laser pulse. Here we show that it is possible to optimize the number of accelerated electrons by adjusting the laser pulse and contrast ratio. For these laser pulses and the aluminium foil target with a thickness of 11 μm, the number of electrons detected at the fluorescent screen reached a maximum when the contrast ratio of the nanosecond amplified spontaneous emission (ASE) worsened from 10^−7^ (Fig. 3, solid circles). The number of electrons was proportional to *E*^0.51^, where *E* is the laser pulse energy on the target. The open circle in Fig. 2a was obtained with the contrast ratio of the nanosecond ASE and laser pulse energy set to approximately 3 × 10^−7^ and 110 mJ, respectively, and then the charge was measured to be 20 fC. In this case, the FWHM pulse width was 75 fs.

**Figure 4:**
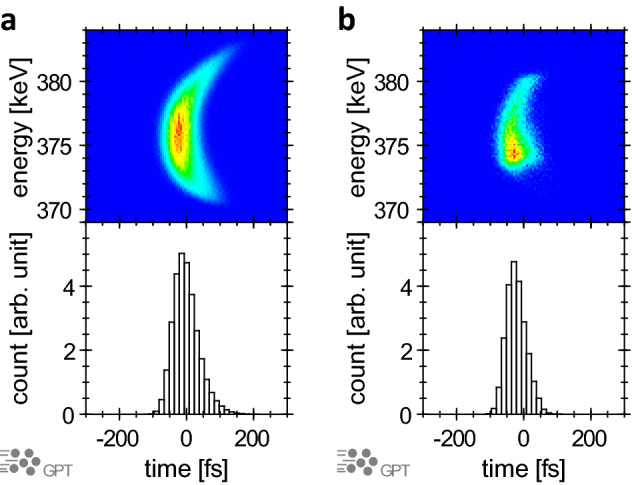
Electron pulse width calculated using the GPT code. (**a**,**b**) Phase-space distribution at the compression point in the absence (**a**) and presence (**b**) of the 50-μm aperture.

Figure 2b shows the measured electron pulse width under the most compressed condition in Fig. 2a (open circle). For this measurement, the FWHM of laser pulse width was 40 fs and the FWHM of the correlation function was 99 fs. Based on the numerical result from Eq. (6) (Fig. 2b), the FWHM of the electron pulse width was determined to be 75 fs. The correlation function has noise at the time delay of 100 to 300 fs. This signal is attributed to electrons emitted from the laser plasma at a delayed timing, and their number is estimated to be 20% of the total number of electrons. Although the electron pulse width using the 50-μm aperture could be obtained experimentally, it was necessary to further evaluate the pulse width including the electrons which are not cut away by the aperture. This estimation was performed using the GPT code (Fig. 4). In this calculation, the optics for the electrons included the solenoid magnetic lens with a 1-mm-diameter aperture, dipole magnets with a slit, and quadrupole magnets, thereby reproducing the experimental equipment. The initial pulse width of the electron pulse was set to 49 fs (FWHM) with a Gaussian profile, which were selected to reproduce the experimentally obtained pulse width at the compression point of 75 fs (FWHM). Although the pulse width of the laser that accelerated the electron pulse was 40 fs in our experiments, the GPT calculations were not in agreement with the experimental results unless the initial electron pulse width was increased to 49 fs; when the initial electron pulse width was set to 40 fs, the electron pulse width at the compression point decreased to 69 fs. The reason for this cannot be explained solely by the aberration of the optics, and the interaction between the electrons and laser plasma in the acceleration phase is also considered to play a role. Although it is impossible to accurately measure the pulse width immediately after acceleration, it is thought that electron pulses with a width similar to that of the laser pulses should be generated^57^. Owing to the interaction between the electron pulses and the high-density and high-energy plasma, we believe that the electron pulse may have been subjected to an incompressible chirp. The upper and lower panels of Fig. 4 show the energy distributions of the electron pulses and the corresponding histograms for the electrons in the presence and absence of the aperture. Without the aperture, non-linear chirp, which cannot be compensated for using the dipole magnet compressor, broadened the electron pulse width. With the aperture, the uncompressed electrons were eliminated to afford a shorter electron pulse. The pulse widths with and without the aperture were 75 and 89 fs (FWHM), respectively. From the above, we can conclude that an electron pulse was obtained with an FWHM pulse width of 89 fs and a charge of 16 fC.

### Long-term timing jitter stability

**Figure 5:**
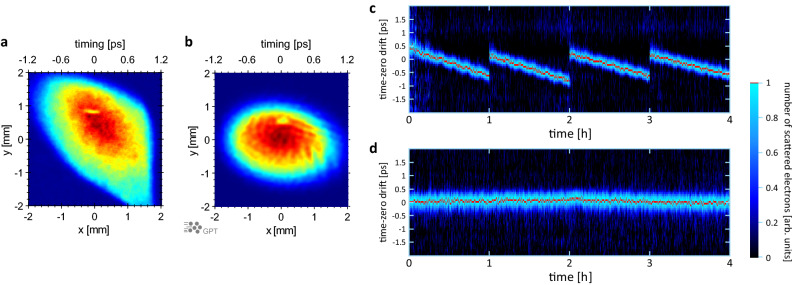
Time-zero drift measurement. (**a**,**b**) Images of an electron pulse on the fluorescent screen with the imaging lens obtained via experiment (**a**) and simulation (**b**). The electron lens relays the image 26 mm behind the compression point, where the electron pulse and laser pulse intersect, onto the fluorescent screen. The shadow observed at [*x*, *y*] = [0 mm, 0.8 mm] in (**a**) corresponds to the part that was scattered by the intense laser pulse. In (**b**), this shadow appears at [*x*, *y*] = [0 mm, 0.6 mm]. If the intersection time (interaction position) of the laser pulse and electron pulse deviates, this shadow moves in the *x*-axis direction. Hence, the *x*-axis direction in this figure corresponds to the time-zero drift. The drift was calibrated by moving the delay stage, and the time scale is shown at the top of (**a**) and (**b**). (**c**,**d**) Time-zero drift measurements over 4 h, showing the electron density distribution in the *x*-axis direction. The region containing scattered electrons appears in blue, and the red line indicates the peak. (**c**) shows the case where the system was not subjected to temperature control; owing to the excessive drift, we moved the delay stage every hour to correct the position. (**d**) shows the results when the system was subjected to temperature control using a chiller. As shown in Fig. 6a, the RMS timing jitter was 14 fs.

**Figure 6:**
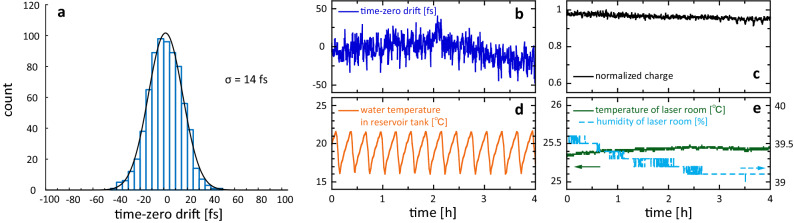
Timing jitter and comparison of the timing drift and environmental parameters. (**a**) Histogram of the time-zero drift with temperature control using the chiller. (**b**) Time-zero drift using the chiller based on the same data as Fig. 5d with the vertical scale expanded. (**c**) Fluctuation in the number of electrons. (**d**) Temperature variation in the reservoir tank of the chiller used to cool the electric stage. (**e**) Temperature and humidity variation of the room containing the laser system.

The time-zero drift of the entire system, which is the pulse-to-pulse timing jitter over a prolonged period, was measured from the ponderomotive scattering of the laser and electron pulses, as shown in Fig. 5. The timing at which the laser pulse and electron pulse intersect was measured over 4 h, and the time-zero drift was measured after removing the 50-μm-diameter aperture. When an electron pulse and a laser pulse intersect at the compression point, a shadow of the laser pulse is created on the electron pulse. A change in the timing of the laser pulse and electron pulse can be observed as a movement of this shadow in the *x*-axis direction (Fig. 1). This shadow of the electrons excluded by the laser was fitted with a Gaussian function, and the centre position was plotted as the time-zero position. The shadow of the laser pulse was transferred to the fluorescent screen using an electron lens with a permanent magnet at double magnification and a resolving power of 16.7 fs/pixel. The red lines in Fig. 5c show the results when the system was not subjected to temperature control, revealing a large timing drift in one direction. Owing to this excessive drift, we moved the delay stage every hour to correct the position. All the experimental equipment was installed inside a vacuum chamber. The large drift was attributable to the heat generated by the motor-driven stages and the quadrupole magnets, which was estimated to be several watts. This heat was removed by introducing cooled water into the vacuum chamber, which dramatically suppressed the large drift, as shown in Fig. 5d. The resulting RMS timing jitter was extremely small at only 14 fs (Fig. 6a), which to our knowledge is the lowest value reported to date. The RMS time resolution was then $\sqrt {\left( {\text{38 fs}} \right)^{{2}} + \left( {\text{14 fs}} \right)^{{2}} } = {\text{40 fs}}$, demonstrating that this system exhibited extremely high temporal resolution. Figure 6c shows the fluctuation of the number of electrons with respect to time. The RMS fluctuation in the number of electrons was 1.3% and it remained stable over a long period. The timing jitter of 14 fs originated from two sources. The first was the resolution of the imaging system composed of the electron lens and fluorescent screen, which can be observed as the short-duration fluctuations in the present time-zero drift measurement (Fig. 6b). The time resolution could be improved by increasing the magnification factors of the electron imaging system that transfers the image to the fluorescent screen and the optical lens attached to the CCD camera, which should reduce the timing jitter. The second source was external perturbation, such as fluctuations in the water temperature, room temperature, or humidity. Figure 6d shows the water temperature in the reservoir tank of the chiller used to cool the motor-driven stages and quadrupole magnets. The timing drift of the electron pulse was rather stable even with extremely rough temperature control. However, variation in the time-zero drift was observed over several hours, as shown in Fig. 6b. This may be attributable to fluctuation of the average temperature in the reservoir tank or the temperature or humidity of the room. Our system had no active feedback system for stabilizing laser pointing. Slight variations of the laser path due to fluctuations induced by changes in the temperature and humidity of the room containing the laser system may have adversely affected the time-zero drift (Fig. 6e). The RMS time-zero drift was estimated to be approximately 8.7 fs for the slow-changing component and 11 fs for the short-duration component. Although the long-term variation of the time-zero drift is sufficiently small for the electron pulse width, its origin has not been identified and achieving finer control is a challenge for the future.

### 100-fs time step backlight imaging with ultrafast electron pulses

**Figure 7:**
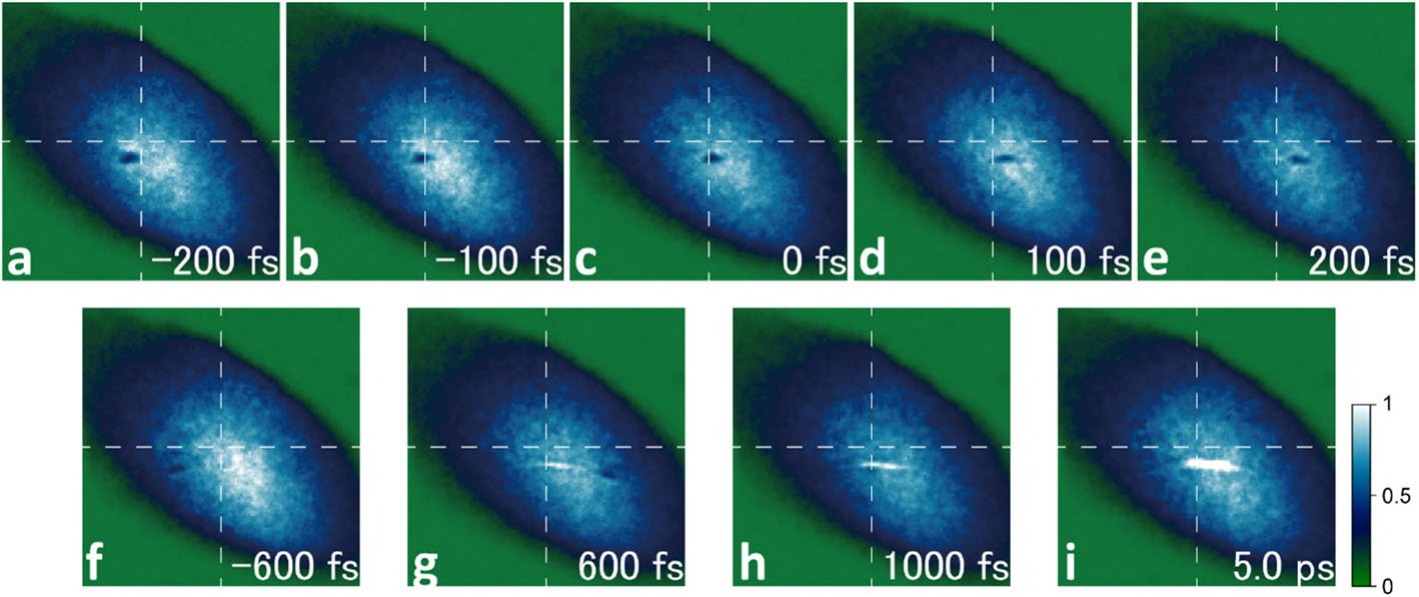
Electron-backlight images of an intense laser pulse propagating in a vacuum and the laser-induced plasma. (**a**–**i**) Snapshots of the shadow picture of the intense laser pulse with the electron pulses at 100-fs time steps (**a**–**e**) and − 600 fs, 600 fs, 1 ps, and 5 ps after passing of the laser pulse (**f**–**i**) obtained using the electron pulses. The experimental setup used to obtain these images was the same as that used to measure the timing jitter. Each snapshot is an average of 10 shots. The propagation of the laser pulse was measured in a vacuum chamber with a pressure of 5 × 10^−2^ Pa. The laser pulse interacted with the small amount of residual gases inside the vacuum chamber to create low-density plasma, as observed in (**g**–**i**). See the Supplementary Video for the motion of the laser pulse from − 1 to 5 ps with a time step of 100 fs.

To demonstrate the capability of the ultrafast electron pulses, we used them to capture the propagation of the intense laser pulse and visualize the plasma generated by the laser pulse with a time resolution of 100 fs. The results are presented as an animation in the Supplementary Video file. The experimental setup was the same as that used to measure the time-zero drift. The electron pulse was an almost parallel beam with a diameter of approximately 2 mm and intersected the laser pulse at the compression point. The intense laser pulse, which was semi-circular with a diameter of 50 mm, was focused by an off-axis parabolic mirror with a focal length of 165 mm. The timing at which the electron pulse and laser pulse intersected was controlled by the delay stage, and images were acquired at 100-fs time steps as presented in Fig. 7a–e. Using this method, we succeeded in clearly capturing the propagation of the laser pulse at 100-fs intervals. Figure 7f–i show the images obtained 600 fs before and 600 fs, 1 ps, and 5 ps after the laser pulse passed through the compression point. The animation in the Supplementary Video shows the temporal and spatial development of the laser pulse and laser-generated plasma when the timing of the laser pulse arrival was changed from − 1 to 5 ps with a time step of 100 fs. In this animation, we can observe how the laser pulse is “focused”, that is, concentrated from a large diameter to the focal spot size, and then diffused to the larger diameter. At a distance of more than 150 µm from the compression point (time delay $\left| {{\Delta }\tau } \right| >$ 500 fs), the laser pulse appears to be divided into two parts, namely, an upper part and a lower part (Fig. 7f,g). The near-field pattern of the laser beam exhibited deviations with strong intensity distributions at the top and bottom of the pattern to afford the shadow images shown in Fig. 7f,g. After the intense laser had passed through, extremely dilute plasma was generated around the laser focus position, as revealed by the linear bright area observed in the electron pulse in Fig. 7g–i. This bright line appeared when an electron pulse crossed the plasma and experienced a force towards the centre of the plasma. The pressure inside the vacuum chamber was 5 × 10^−2^ Pa, corresponding to approximately 10 particles µm^−3^. When a high-intensity laser was focused, its intense electric field stripped off the electrons of the particles remaining in the vacuum, generating a plasma with a particle density of ~ 1 × 10^13^ cm^−3^ distributed over the Rayleigh range of the intense laser pulse. Some of the stripped electrons will be ejected from the focused portion of the laser pulse to a sufficiently long distance^64–66^. Consequently, it is considered that the electron trajectory was bent by the sheath electric field generated by the plasma or the electric field generated by the locally existing non-neutral plasma, creating the bright line observed on the screen. The process by which this bright line developed was roughly evaluated using the GPT code and could be reproduced by the electric field generated by a cylindrical charge having a positive charge of approximately several picocoulombs. Further clarification would require calculation of the electric field due to plasma creation using PIC code or the like, although it can be concluded that the current method successfully provides an exceedingly stable and ultrashort electron pulse suitable for capturing ultrafast phenomena.

## Conclusions

In summary, we have successfully developed an ultrashort electron pulse source using fast electrons accelerated by irradiation of a thin aluminium foil with an intense femtosecond laser. The RMS pulse width of the obtained electron pulse was extremely small at only 89 fs at FWHM. As there is essentially no mechanism for generating timing jitter or long-term timing drift in the proposed pulse compression system, the electron pulses were extremely robust in terms of time fluctuations. This very small timing jitter was achieved by incorporating a suitable heat-removal mechanism, and the RMS timing drift was extremely stable at only 14 fs. We anticipate that the proposed system will be useful as an electron source for observing ultrafast phenomena, such as ultrafast electron diffraction and laser-induced electromagnetic fields, and make a significant contribution to future ultrafast science research.

## Methods

### Laser system

The intense laser pulses for accelerating the electron beam and measuring the electron pulse width and timing jitter were obtained by splitting a laser pulse generated by a Ti:sapphire CPA laser system^47,48^. This system comprises three multi-pass amplification stages. The pulses from the oscillator (Vitara-T HP, Coherent) were amplified with an eight-pass pre-amplifier and two five-pass power amplifiers. After four passes in the pre-amplifier, a nanosecond Pockels cell system (Model 5046SC, FastPulse Technology) was used to chop an 80-MHz pulse train from the oscillator. After the pre-amplifier, a sub-nanosecond Pockels cell system (UPC-088, Leysop) was used to reduce the nanosecond ASE. The laser intensity contrast ratio was approximately 10^−6^ at 2 ps before the peak of the main laser pulse and approximately 10^−7^ for the nanosecond ASE, whereupon the input power into the pre-amplifier was 340 mW. To adjust the pulse contrast ratio, the pulse energy of the chirped pulses after the pulse stretcher was attenuated using neutral density filters. The output energy from the power amplifiers was kept constant, and only the gain for the ASE was changed. In the present experiments, the input power into the pre-amplifier with the neutral density filters was 30 or 110 mW. The input energies of 30 and 110 mW correspond to changing the contrast ratio by 11-fold and 3.1-fold compared with the contrast ratio at an input energy of 340 mW. A plasma mirror with a reflectivity of 10^−2^ was used to reduce the ASE level. The plasma mirror reduced the ASE level from 10^−6^ to 10^−8^ at 2 ps before the peak and from 10^−7^ to 10^−9^ for the nanosecond ASE.

The laser pulse from the compressor with a diameter of 50 mm was split in half by a gold mirror inside the vacuum chamber. Each laser pulse fired at the target foil and at the compression point was focused using an off-axis parabolic mirror with a focal length of 165 mm. The pointing stability of the laser pulse, which is critical for the timing jitter owing to its influence on the path length of the laser pulse and electron pulse, was sufficiently high. The RMS pointing stability was within 1.5 μrad in 1 h, corresponding to 0.25 μm in the focal plane.

### Electron pulse width measurement based on ponderomotive scattering

The electron pulse width was measured using the interaction between the laser pulse and electron pulse via the ponderomotive force^20,34,60–63^. At the compression point (Fig. 1a), the electron pulse intersected the laser pulse with a size of 6 × 12 μm at FWHM, a pulse width of 40 fs at FWHM, and an intensity of approximately 1 × 10^18^ W cm^−2^ at a crossing angle of 55°. To improve the temporal resolution, the electron pulse was cut off using a 50-μm pinhole at 25 mm prior to the compression point. The electrons that overlapped the laser were scattered in the *y*-axis direction by the ponderomotive force. By measuring the dependence of the scattered electrons on the time delay τ, the correlation function S(τ) determined from the widths of the electron and laser pulses could be obtained^20,60^. This correlation function is expressed as the integral of the product of the scattered distance *Y* of the deflected electrons on the screen and their density *D*(*X*, *Y*) when the electrons were scattered by the laser pulse.

1 $$ S = \int dXdY\left[ {\left| Y \right| \cdot D\left( {X, Y} \right)} \right] $$

2 $$ \begin{aligned} X & = T\frac{1}{{\gamma m_{e} }}\int dp = \frac{T}{{\gamma m_{e} }}\int dtF_{x} \left( {{\varvec{r}}\left( t \right),t} \right), \\ Y & = T\frac{1}{{\gamma m_{e} }}\int dp = \frac{T}{{\gamma m_{e} }}\int dtF_{y} \left( {{\varvec{r}}\left( t \right),t} \right) \\ \end{aligned} $$

where *X* and *Y* represent the coordinates with respect to the origin of the centre of the electron pulse that reached the screen; *T*, *γ*, m_e_, *p*, and ***r*** are the kinetic energy, Lorentz factor, mass, momentum, and coordinates of the electrons, respectively; and *F*_*x*_ and *F*_*y*_ are the ponderomotive forces. *S*(*τ*) for each time delay $\tau$ is given by

3 $$ S\left( \tau \right) = \int dXdY\left[ {\left| Y \right| \cdot D\left[ {X,Y} \right]} \right] = \frac{T}{{\gamma m_{e} }}\int dtdxdydz\,n_{e} \left( {x, y,z - vt} \right) \times \left| {F_{y} \left( {{\varvec{r}}\left( {t, \tau } \right),t} \right)} \right| $$

where $\tau$ is time delay, *v* is the electron velocity, and $n_{e} \left( {x, y,z - vt} \right)$ is density distribution of the electron pulse. If the pinhole is sufficiently smaller than the electron beam size, then the transversal distribution of the electron pulse is uniform. Assuming that the temporal distribution of the electron pulse is Gaussian, the electron density distribution is given by

4 $$ n_{e} \left( {x,y,z - vt} \right) \propto \left\{ {\begin{array}{*{20}l} {\exp \left( { - \left( {\frac{z - vt}{{\sqrt 2 v\tau_{e} }}} \right)^{2} } \right), } \hfill & { \left| x \right| \le \frac{\varphi }{2} ,\left| y \right| \le \frac{\varphi }{2} } \hfill \\ {0, } \hfill & { \left| x \right| > \frac{\varphi }{2} ,\left| y \right| > \frac{\varphi }{2} } \hfill \\ \end{array} } \right. $$

where *φ* is the electron beam size, *τ*_*e*_ is the RMS electron pulse width, and *v* is the electron velocity. The temporal and spatial distributions of the laser pulse are Gaussian, so the intensity *I* of the laser pulse is

5 $$ I\left( {{\varvec{r}}\left( {t, \tau } \right),t} \right) \propto \exp \left( { - \left( {\frac{ - z\sin \theta + x\cos \theta - ct + c\tau }{{\sqrt 2 c\tau_{l} }}} \right)^{2} - \left( {\frac{y}{{w_{y} }}} \right)^{2} - \left( {\frac{z\cos \theta + x\sin \theta }{{w_{z} }}} \right)^{2} } \right) $$

where *c* is the speed of light, τ_l_ is the RMS laser pulse width, *θ* is the crossing angle between the laser pulse and electron pulse, and *w*_*y*_ and *w*_*z*_ are the beam size of the laser pulse in the *y*- and *z*-directions. Then, the correlation function *S*(*τ*) can be expressed as

6 $$ \begin{aligned} S\left( \tau \right)  \propto \int dtdxdydz\,n_{e} \times \left| {F_{y} \left( {{\varvec{r}}\left( {t, \tau } \right),t} \right)} \right| = \int dtdxdydz\,n_{e} \times \left| {\frac{\partial }{\partial y}I\left( {{\varvec{r}}\left( {t, \tau } \right),t} \right)} \right| \\  \propto \mathop \int \limits_{ - \infty }^{\infty } dt\mathop \int \limits_{{ - \frac{\varphi }{2}}}^{{\frac{\varphi }{2}}} dx\mathop \int \limits_{ - \infty }^{\infty } dz\exp \left( { - \left( {\frac{ - z\sin \theta + x\cos \theta - ct + c\tau }{{\sqrt 2 c\tau_{l} }}} \right)^{2} } \right) \\  \quad \times \exp \left( { - \left( {\frac{z\cos \theta + x\sin \theta }{{w_{z} }}} \right)^{2} } \right)\exp \left( { - \left( {\frac{z - vt}{{\sqrt 2 v\tau_{e} }}} \right)^{2} } \right) \\ \end{aligned} $$

**Figure 8:**
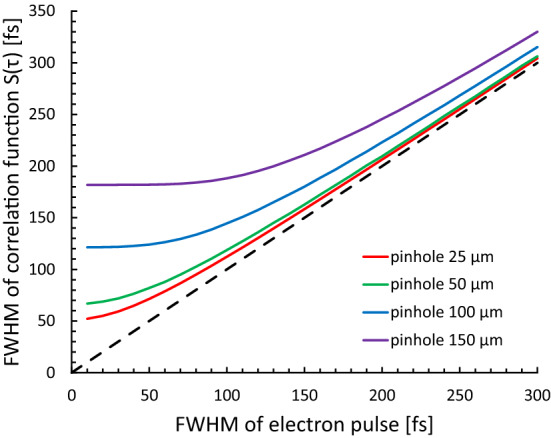
Pulse width of the correlation function with respect to the electron pulse width for a laser pulse width of 40 fs. The lines are calculated from Eq. (6) for pinhole diameters of 25, 50, 100, and 150 µm.

The original electron pulse width was inferred by comparing the numerical results from Eq. (6) with the experimental results. In the present experiments, the correlation function obtained was fit by a Gaussian function with an RMS width of τ_s_. Our correlation function measurements required that the spatial size of the electron pulse be limited by a pinhole to achieve sufficient temporal resolution because the lateral beam size of the electron pulse is larger than the region where the electron pulse and laser pulse interact. We determined the appropriate pinhole size based on Eq. (6). Figure 8 shows the calculation result from Eq. (6) for various pinhole sizes and a laser pulse width of 40 fs. When the size of the pinhole is 50 µm, if the electron pulse width is ≥ 50 fs, the calculated change in the electron pulse width can be detected as a change in the width of the correlation function.

## Supplementary information

Supplementary Video.
